# Very Rare Presentation of Cerebrovascular Accident in 20-Year-Old Man With Familial Mediterranean Fever—Case Report

**DOI:** 10.1177/1179547617749208

**Published:** 2018-01-03

**Authors:** Miramir Aghdashi, Seyed-Mostafa Seidmardani, Sara Vossoughian, Seyed Arman Seyed Mokhtari

**Affiliations:** 1 Department of Rheumatology, Urmia University of Medical Sciences, Urmia, Iran; 2 Department of Internal Medicine, Urmia University of Medical Sciences, Urmia, Iran; 3 Student Research Committee, Urmia University of Medical Sciences, Urmia, Iran

**Keywords:** Familial Mediterranean fever, central nervous system, cerebrovascular accident

## Abstract

Familial Mediterranean fever (FMF) is characterized by recurrent episodes of fever accompanied by serosal, synovial, or cutaneous inflammation. The central nervous system (CNS) is rarely involved in FMF. The CNS involvement includes demyelinating lesions, posterior reversible encephalopathy syndrome, pseudotumor cerebri, optic neuritis, and cerebral vasculitis. Here, we present a 20-year-old man, a known case of FMF with abrupt left-sided hemiparesis. Brain magnetic resonance imaging revealed right periventricular infarction. Normal echocardiography ruled out cardioembolism, and thrombophilia workup was negative. Therefore, FMF-induced cerebrovascular accident was considered. Although rare, CNS involvement as a result of FMF disease should also be considered when encountering patients with FMF and CNS manifestations.

## Introduction

Familial Mediterranean fever (FMF) is an inherited disease characterized by recurrent episodes of fever in the course of serosal, synovial, or cutaneous inflammation. It may eventually lead to systemic amyloidosis in some individuals. The FMF gene encodes a 781-amino acid, 95-kDa protein called pyrin (or marenostrin). It regulates caspase-1 interleukin 1 (IL-1)-converting enzyme through mechanisms such as interaction between pyrin and an intermediary adaptor protein, leading to IL-1 secretion. Pyrin is also expressed in eosinophils, granulocytes, dendritic cells, and monocytes and also in synovial and peritoneal fibroblasts.^1^

Neurologic involvement rarely occurs in FMF but instances of pseudotumor cerebri, optic neuritis, and recurrent aseptic meningitis have been reported. Association between FMF and CNS complications of polyarteritis nodosa (PAN), Henoch-Schonlein purpura, and Behçet disease has also been reported. Ischemic stroke, cranial nerve lesions, and demyelinating lesions are the other CNS involvements which could occur in patients with FMF. Among them, CNS demyelination is probably the most frequent finding. Whether CNS involvement is a direct manifestation of FMF or is solely a coincidental event has not yet been understood.^2^

Here, we have presented an old case of FMF with recent development of CNS involvement.

## Case Presentation

**Figure 1.**
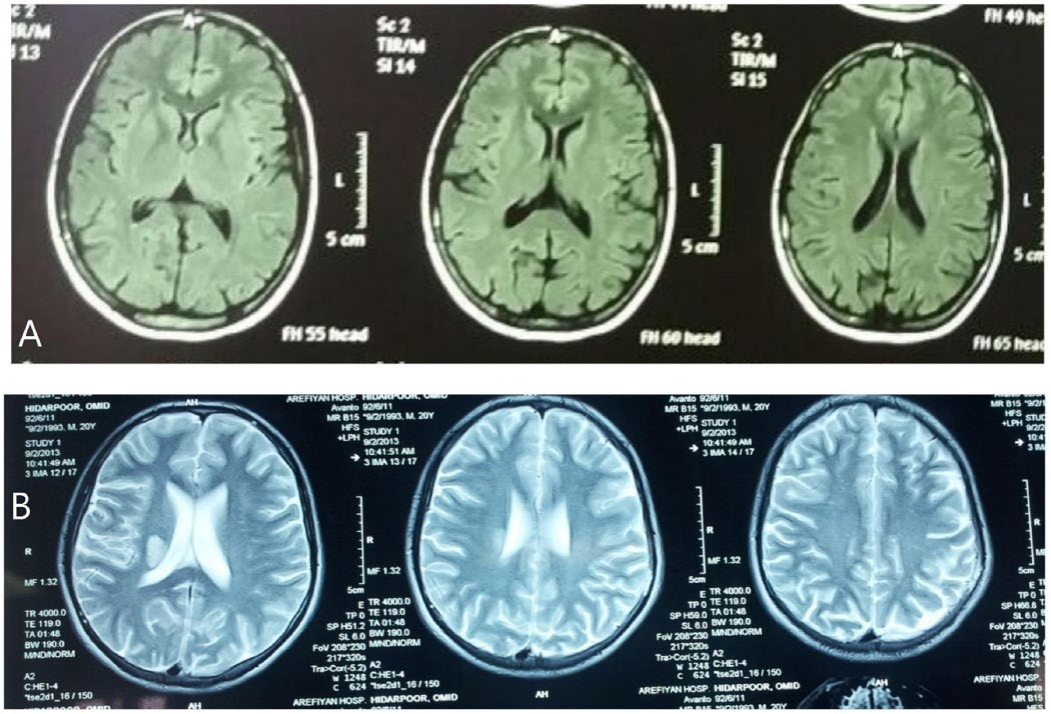
Brain magnetic resonance imaging of the patient showing (A) no abnormalities and (B) infarction of right periventricular region with extension to adjacent basal ganglia.

A young 20-year-old known case of FMF since 17 years was admitted due to abrupt left-sided paresthesia and hemiparesis that had begun 2 weeks before admission. Sudden and intermittent anesthesia of the left side of the face and anesthesia and paresis of the left hand and foot which lasted for 10 minutes were the first symptoms experienced. Several attacks happened in the course of a day and the most recent lasted for 24 hours, leading him to seek medical help. The patient was hospitalized in another center. He complained of a simultaneous fever at the onset of attacks. However, no fever was detected during his stay at the hospital. Paresis did not occur following the second day of hospitalization and all neurologic complaints were completely resolved. A brain magnetic resonance imaging (MRI) revealed evidence of ischemic infarction in the right periventricular region with mild extension to adjacent basal ganglia, whereas there are no abnormalities in brain MRI of patient 10 days before hospitalization (Figure 1). He was discharged after 10 days and referred to Imam Khomeini Hospital for further evaluations.

The patient had no neurologic complaints on admission. No previous occurrence of visual loss, aphasia, or loss of consciousness was noticed. No oral aphthous ulcers were observed; however, the occurrence of a recent metronidazole-related genital aphthous ulcer was claimed which had resolved after drug discontinuation.

The patient is known case of FMF with homozygous mutation on M694V since 17 years, receiving colchicine therapy once daily. An episode of jaundice with elevated level of liver enzymes had happened. Treatment with ursobil once daily resulted in remission. He had undergone abdominal surgery twice because of severe abdominal pain resembling acute abdomen. However, no abdominal pathology other than serous inflammation, possibly due to FMF, was found in surgically obtained specimens.

The patient was single and living with his parents. No history of smoking, alcohol consumption, or use of illicit drugs was given.

At the admission to the hospital, physical examination revealed a blood pressure of 90/60 mm Hg, pulse rate of 88, respiratory rate of 10, and body temperature of 36.5°C. No cardiac or lung abnormalities were observed in examination. No lesions were found in skin examination. No abdominal tenderness was detected. The liver span was about 16 cm on the midclavicular line, and the spleen was almost not palpable under costal margin. No bruit was heard in auscultation of carotid and renal arteries. Neurologic and musculoskeletal examinations were thoroughly normal. Measured proteinuria in 24-hour urine was 79 mg/24 hours. A mild leukocytosis (13 400 per mm^3^) with neutrophil predominance along with a mild anemia (hemoglobin: 12 g/dL) and raised erythrocyte sedimentation rate (83 mm) was found in his laboratory tests. The values of blood sugar, international normalized ratio, partial thromboplastin time, electrolytes, renal function test, and liver function test were all normal. C3, C4, and vascular markers including antinuclear antibody (ANA), anti-Smith antibody (anti-Sm Ab), anti-double-stranded DNA (anti-dsDNA), cytoplasmic antineutrophil cytoplasmic antibody, and perinuclear antineutrophil cytoplasmic antibody were present at normal levels. Serum levels of protein C, protein S, antithrombin III, homocysteine, and anticardiolipin Ab (IgG) were also in normal range. Human leukocyte antigen (HLA) B5 was positive.

Echocardiography revealed normal findings with ejection fraction of 60% and abdominal sonography showed increased size of liver and spleen (165 and 155 mm, respectively). Normal hepatoportal flow was observed in portal, hepatic, and suprahepatic veins in color Doppler sonography. Normal flow was observed in carotid artery in color Doppler sonography.

He was discharged on day 10 of admission with prescription of prednisolon, warfarin, and colchicine. No new neurologic complaints or remaining complication from previous attacks was found on the follow-up visit.

## Discussion

Familial Mediterranean fever is an autosomal recessive disease involving multiple systems. North Africans, Armenians, Jewish, Turkish, Druze, and Arabs have a predisposition for FMF. The disease is characterized by recurrent episodes of inflammation occurring in peritoneum, pleura, and synovium causing abdominal pain, pleural effusion, and arthritis. Peripheral nervous system involvement is a common finding. Reduced tendon reflexes and myalgia are the 2 major manifestations of peripheral nervous system involvement. However, involvement of the central nervous system is a rare finding in the patients with FMF. Demyelinating lesions (multiple sclerosis–like lesions), pseudotumor cerebri, and posterior reversible encephalopathy syndrome have also been observed in patients with FMF.^3^ Occurrence of optic neuritis, cerebral vasculitis, and also hypercoagulable state secondary to amyloidosis has also been reported in patients with FMF.^2^

In the present case, evidence, such as MRI results with notable infarction of right periventricular region extended to the adjacent basal ganglia, are in favor of single-hemisphere involvement of the brain. The possibility of cardioemboli, as a major suspect of cerebrovascular accident (CVA), was ruled out by normal echocardiography, and also possibility of carotid dissection as another suspect of cardiovascular accident was ruled out by normal color Doppler sonography. The normal level of serum protein C, protein S, antithrombin III, hemocysteine, and anticardiolipin Ab (IgG) ruled out the existence of any hypercoagulative state in the patient. The normal level of serum ANA, anti-dsDNA, and anti-Sm Ab ruled out systemic lupus erythematous in the patient. By the nephrotic syndrome diagnosis protocol, measured proteinuria in 24-hour urine was 79 mg/24 hours (for nephrotic syndrome approval, 24-hour urine proteinuria must be greater than 150 mg/24 hours) that ruled out the nephrotic syndrome. The patient did not smoke and had no other risk factors, such as hypertension, hyperlipidemia, or positive family history, which could lead to strokes. Hence, it could be concluded that the CVA is related to the persisting FMF disease in the patient.

Kalyoncu et al showed that patients with FMF with homozygote M694V mutations also have endothelial dysfunction. They reported that explained causes are yet to be found in 40% of patients with FMF, occurring with a higher prevalence in younger patients. In their study, 7 of 15 cases with FMF were stricken with CVA. They also did not find the causes, such as cardioembolism or known previous hypercoagulability state, in 5 patients with FMF who had presented with stroke. Only a single patient had a patent foramen ovale with a significant right to left shunt; however, no clues of paradoxical embolism were observed in further investigations.^2^

Kofoed et al proved that fibrinogen is able to predict the possibility of future ischemic strokes in the general population. Meanwhile, fibrinogen is an important marker of FMF activity. The prediction value of fibrinogen in showing stroke possibility, particularly among young and middle-aged men, is considerably high.^4^ In addition to marked inflammatory complications such as serosal, pleural, and synovial involvement, subclinical inflammation also occurs predominantly in the patients with FMF. It is suggested that endothelial dysfunction and resulting strokes in patients with FMF might be caused by subclinical inflammation.^5^

A prominent point of the present case is occurrence of the cerebrovascular accident in his low. It is in good correlation with the pattern reported by Kalyoncu et al. In general, only 20% to 30% of strokes occur under the age of 45 years in developing countries. However, Kalyoncu et al discovered that all of their patients were young (mean age of 28.5 years).^2^ Thus, the occurrence of stroke in the current case with a young age and without any possible explanation other than underlying FMF seems rational.

Association of FMF with Henoch-Schonlein purpura, PAN, and Behçet disease has been observed in the other studies.^2^ Such diseases can raise the incidence of stroke. However, no confirming evidence of the aforementioned diseases were found in the current case. All laboratory tests for collagen diseases were negative and the radiologic features of MRI were in favor of CVA rather than vasculitis. Although HLA B5 was positive in our case, no other characteristics of Behçet disease were observed and his genital aphthous was drug induced. However, the accurate diagnosis of FMF, PAN, or Behçet disease in some cases is really hard.^2^

It has recently been reported that FMF is associated with the occurrence of a hypercoagulable state.^6^ This might be another basis of this inherited disease leading to a raised incidence of CVAs in patients with FMF.

## Conclusions

Although the exact mechanism of relation between FMF and CVA remains obscure, and CVAs are a rare complication in patients with FMF, FMF-related stroke is worth to be considered, at least in challenging cases. The most striking feature is the young age of patients with FMF and CVA. Further studies on the mechanism of such a relationship and the measures necessary to decrease CVA incidence in patients with FMF, such as control of CVA risk factors, are suggested.
